# Analysis of the trend of malaria prevalence in Ataye, North Shoa, Ethiopia between 2013 and 2017

**DOI:** 10.1186/s12936-018-2474-3

**Published:** 2018-09-05

**Authors:** Daniel Getacher Feleke, Daniel Gebretsadik, Angesom Gebreweld

**Affiliations:** Department of Medical Laboratory Science, College of Medicine and Health Sciences, Wollo University, Dessie, Ethiopia

**Keywords:** Malaria, Prevalence, Ethiopia

## Abstract

**Background:**

Malaria is one of the major public health problems worldwide. In Ethiopia, there is a significant decline in disease burden; however, the overall trend of malaria prevalence is not studied or well-documented in different localities. Hence, the initiation of this study was to analyse the 5-year trends of malaria prevalence in Ataye, North Shoa, Ethiopia.

**Methods:**

A retrospective laboratory record review was conducted in Ataye Hospital, North-Shoa, Ethiopia. Malaria data reported from 2013 to 2017 were carefully reviewed from January to March 2018.

**Results:**

A total of 31,810 blood films were prepared and examined from malaria-suspected patients at Ataye District Hospital from 2013 to 2017. Of the examined blood films, 2670 (8.4%) were microscopically confirmed malaria cases. The trend of malaria prevalence in the present study seems non- fluctuating. *Plasmodium falciparum* and *Plasmodium vivax* accounted for 2087 (78.2%) and 557 (20.9%) cases, respectively. From total positive cases, 1.0% of cases were mixed *P. falciparum*/*P. vivax* infections, and that no *Plasmodium malariae* and *Plasmodium ovale* infections were found by malaria microscopists. Malaria cases were higher in males 1584 (5.0%) than females 1086 (3.4%). With regard to age groups, higher numbers of malaria cases were observed in age group 15–45 years old. Malaria cases were high in spring (September to December), which is a peak malaria transmission period in Ethiopia.

**Conclusion:**

Malaria is still among the major public health problems in the country. *P. falciparum* is the dominant species in the study area followed by *P. vivax*. Enhancing malaria detection and speciation skill of laboratory personnel and scaling up malaria control and prevention activities are very crucial to significantly reduce the burden of malaria in the study area.

## Background

Malaria is one of the major public health problems worldwide and it causes significant mortality and morbidity in the least developed areas of tropical Africa, Asia and Latin America [1]. In 2016, there were an estimated 216 million malaria cases worldwide, 90% of those cases were in African region followed by South-East Asia Region (7%) and Eastern Mediterranean Region (2%) [2].

In Ethiopia, 75% of the areas are malarious and an estimated 68% of the population lives in these areas [3–5]. Malaria transmission exhibits a seasonal and unstable pattern in Ethiopia, with transmission varying with altitude and rainfall [6]. The peak malaria transmission season in the country is from September to December, following the main rainy season from June/July to September. *Plasmodium falciparum* and *Plasmodium vivax* are the most dominant Plasmodium species in Ethiopia [5, 7].

Ethiopia guided its malaria prevention and control activities using the national strategic plan to reduce the burden of malaria significantly. Early diagnosis and prompt treatment, selective vector control that involves use of indoor residual spraying (IRS), insecticide-treated mosquito nets (ITNs) and environmental management are the four major intervention strategies that are being applied in Ethiopia [8].

In Ethiopia, there is a significant decline in disease burden; however, the overall trend of malaria prevalence is not studied or well-documented in different localities. Malaria prevention and control activities are intensified in Ethiopia by Ministry of Health, NGOs working on malaria and other stakeholders. Therefore, estimating malaria incidence and time trends is very important for the expansion of intervention strategies or to design new ones to tackle the disease since this information has significant input for combating malaria. Ataye is one of the hot areas in Ethiopia and it is malarious area. Ataye town has governmental (one health centre and one hospital) and non-governmental health facilities. Hence, the initiation of this study was to analyse the 5-year trends of malaria prevalence in Ataye, North Shoa, Ethiopia.

## Methods

### Study area

Ataye is a populated place in Amhara Regional State. It is located at an elevation of 1468 metres above sea level. Ataye district is one of the 22 districts found in Northern Shoa around 280 km away from the capital of Ethiopia, comprising a total population of 125,914 [9]. Ataye is one of the hot areas in Ethiopia and commonly affected by Plasmodium species and intestinal parasites. The town has governmental (one health centre and one hospital) and non-governmental health facilities. The health centre was the only governmental health facility that gives service for the patients in the town. By considering the patient flow and variety of cases the District Hospital was founded in January 2013 besides the health centre. This retrospective study was conducted at Ataye District Hospital from January to March 2018.

### Study design

This retrospective laboratory record review study carried out to determine 5-year (2013–2017) malaria cases.

### Data collection and data analysis

Five-year malaria data (from January, 2013 to December, 2017) was collected from Ataye hospital laboratory record books from January to March 2018. The hospital was established in 2013 and microscopy was the only and the golden standard diagnostic method for the detection and species identification of Plasmodium parasites. The study hospital strictly follows the standard operating procedures (SOPs) in all phases of the quality control. In Ethiopia, all hospitals and health centres follow a standard operating procedure (SOP) for capillary blood sample collection, smear preparation, staining and blood film examination for malaria parasite detection throughout the country. In the study hospital, thin smears were considered positive for malaria if one or more malarial parasites were seen; and, negative if no asexual form of Plasmodium was observed in 200 high-power fields. On the other hand, thick blood films were taken as positive if one or more malaria parasites have been observed; and, negative if no parasites were seen after examining 1000 white blood cells [10].

When Ataye hospital becomes functional in 2013, it was well equipped and fulfilled with skilled health service providers. The laboratory was also well equipped and had well trained laboratory personnel. For all these reasons, there is a comprehensive and consistent malaria data in the hospital laboratory record books. However, there were some incomplete registrations and those data were excluded. In this record review 7 data were excluded due to incompleteness of factors such as age and sex. All of the laboratory personel in the study hospital had more than 3 years experience in malaria microscopy; they had also additional training on malaria microscopy at least once in their career. Any patients found to be infected with *P. falciparum* were treated with Coartem^®^, and patients infected with *P. vivax* were treated with chloroquine according to national malaria treatment guidelines.

Data were first entered into Excel and then imported into SPSS version 20 (SPSSINC, Chicago, II, USA). To compare the trend of malaria prevalence in male and female and also between age groups Chi square test was used. In all comparisons, P < 0.05 was considered as statistically significant.

## Results

A total of 31,810 blood films were prepared and examined from malaria-suspected patients at Ataye District Hospital from January 2013 to December 2017. Of the examined blood films, 2670 (8.4%) were microscopically confirmed malaria cases. In 2016, higher number of malaria-suspected patients (n = 8066) were examined and 863 (10.7%) of them were microscopically confirmed cases. On the other hand the least number of cases, n = 358 (5.8%) were recorded in 2017. Generally, malaria cases showed an increment from 2013 to 2016 where as there was a decrease in malaria cases in 2017. There were 505 more cases recorded in 2016 compared to the number of malaria cases in 2017. With regard to Plasmodium species, *P. falciparum* and *P. vivax* single-species infections accounted for 2087 (78.2%) and 557 (20.9%) cases, respectively. There were only 26 (1.0%) *P. falciparum* and *P. vivax* mixed infections. The maximum number of mixed infections was observed in 2015. Throughout the reviewed period, the numbers of *P. falciparum* cases were dominant over *P. vivax* with the exception of the year 2017 (Table 1).

**Table 1:** Malaria case distribution in Ataye hospital from 2013 to 2017

Year	Total no. of blood film**s** examined	Total number of cases n **(**%)	*Plasmodium falciparum* n (%)	*Plasmodium vivax* n (%)	Mixed (Pf + Pv)
2013	4853	384 (7.9)	292 (76.0)	88 (22.9)	4 (1.0)
2014	6023	425 (7.1)	338 (79.5)	82 (19.3)	5 (1.2)
2015	6682	640 (9.6)	570 (89.1)	61 (9.5)	9 (1.4)
2016	8066	863 (10.7)	710 (82.3)	147 (17.0)	6 (0.7)
2017	6186	358 (5.8)	177 (49.4)	179 (50.0)	2 (0.6)
Total	31,810	2670	2087 (78.2)	557 (20.9)	26 (1.0)

**Fig. 1:**
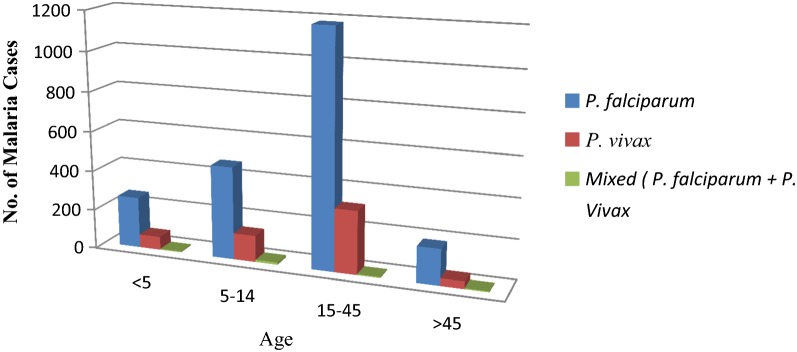
Five years period malaria trends by age at Ataye District Hospital, Ataye, Ethiopia from January, 2013 to December, 2017

Malaria trends with regard to age showed that *P. falciparum* and *P. vivax* cases were higher in age group 15–45 years old. This difference was not statistically significant (P-value = 0.064). Higher numbers of *P. falciparum* and *P. vivax* cases were seen in the 5–14 and 15–45 age categories when compared to the under 5 and over 45 age categories (Fig. 1).

**Fig. 2:**
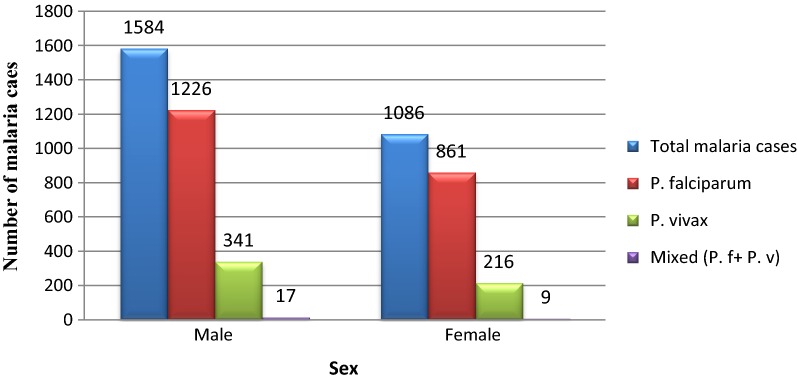
Five years period malaria trends by sex at Ataye District Hospital, Ataye, Ethiopia from January, 2013 to December, 2017

Malaria infections were higher in males 1584/31,810 (5.0%) than females 1086 (3.4%). This difference was statistically significant (P-value = 0.006). With regard to Plasmodium species, *P. falciparum* was the dominant species in males with 1226/1584 (77.4%) while *P. vivax* was higher in females with 861/1086 (79.28%). Similarly, mixed infections were also slightly higher in males than females (Fig. 2).

**Fig. 3:**
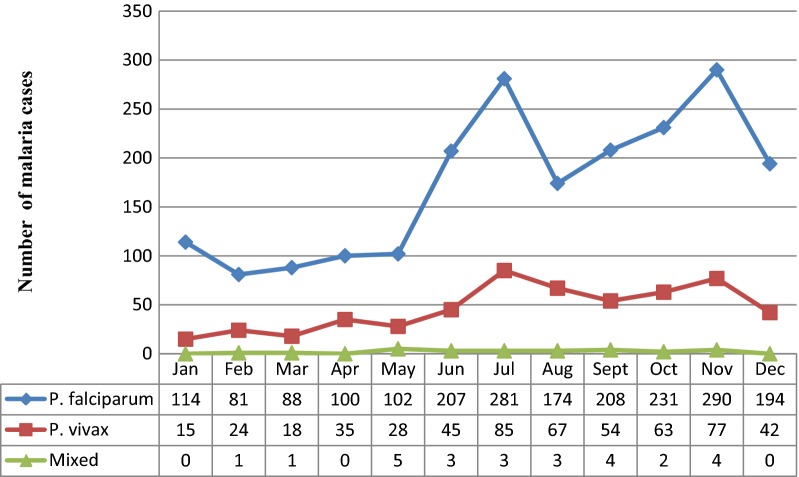
Five years period malaria trends by month at Ataye District Hospital, Ataye, Ethiopia from January, 2013 to December, 2017

Among the factors that affect malaria transmission, seasonal variation has direct role. In the present study, malaria cases were increased from September to December. This period is considered as the peak malaria transmission period in Ethiopia after the heavy rain in July and August. Similarly, there was an increment of malaria cases from May to July (Fig. 3).

**Fig. 4:**
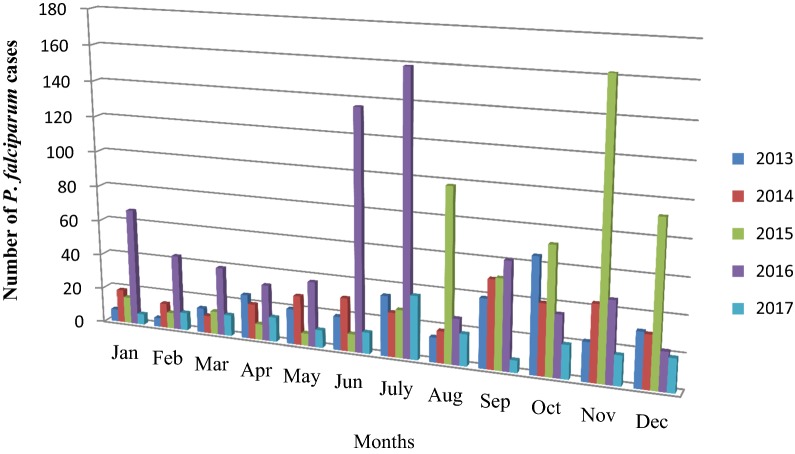
Five years period *Plasmodium falciparum* trends by month at Ataye District Hospital, Ataye, Ethiopia from January, 2013 to December, 2017

*Plasmodium falciparum* cases distribution by months showed a fluctuating trend. However, high numbers of cases were recorded from September to December. *P. falciparum* cases were reach peak in June and July of 2016. In the month of September, *P. falciparum* cases showed a steady increase from 2013 to 2016 and cases were reduced in 2017. There was also high number of *P. falciparum* cases in November and December 2015 (Fig. 4).

**Fig. 5:**
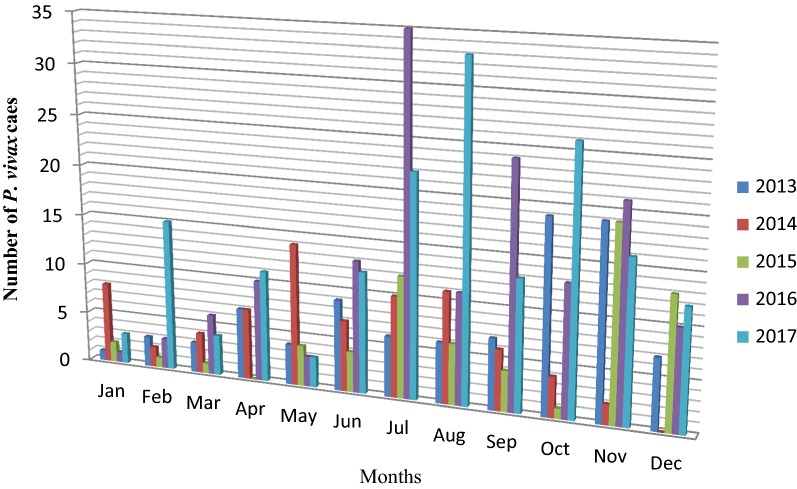
Five years period *Plasmodium vivax* trends by month at Ataye District Hospital, Ataye, Ethiopia from January, 2013 to December, 2017

Similarly, the distribution of case counts of *P. vivax* by month for each year showed that number of cases was high from September to December. There were no *P. vivax* cases in April 2017 December 2014. Higher number of *P. vivax* case was reported in July 2016 and August 2017 (Fig. 5).

## Discussion

In Ethiopia, 75% of the areas are malarious and an estimated 68% of the population lives in these areas [3–5]. The peak malaria transmission season in the country is from September to December, following the main rainy season from June/July to September. Although the malaria prevalence was not studied before, Ataye town is one of the malarious areas in Amhara region. In the present study microscopically confirmed cases were 2670 (8.4%) in Ataye hospital from 2013 to 2017. The minimum and maximum number of cases was recorded in 2013 and 2017, respectively. In this study, the confirmed malaria cases was higher than reported from other similar retrospective studies conducted in Bahirdar city 740 (5.0%) and Kombolcha town (2066, 7.52%), Ethiopia [3, 11]. However, the observed number of malaria cases was lower than reported [12, 13]. The observed variation might be due to difference in climate, altitude variation, laboratory personel skill in malaria parasite detection and community awareness about malaria transmission and control. In the study area, *P. falciparum* was the dominant Plasmodium species which accounted for 2087 (78.2%) of the reported cases in 5 years. This was slightly higher than a result reported from a retrospective study reported in Kola Diba health centre (75%) [13].

Malaria prevalence trend in the present study seems non-fluctuating as microscopically confirmed cases showed a steady increase from 2013 to 2016 for 4 consecutive years. However, the number of malaria cases was decreased in 2017. This was not in agreement with similar retrospective studies in Ethiopia [3, 11, 13]. With the exception of the year 2017 when the number of recorded *P. vivax* cases was higher 179/358 (50.0%) than *P. falciparum* cases, in other reviewed years *P. falciparum* cases dominated. This was more or less in agreement with previous studies [3, 11, 13]. This showed the life-threatening Plasmodium species; *P. falciparum* is the dominant species in the study area and other parts of the country. The finding was also in line with Ethiopian Ministry of Health report [5].

The maximum number of malaria cases in 2016 might be due to in consistent implementation of malaria prevention and control strategies in the study area. Compared to other reviewed years, the number of malaria-suspected patients and microscopically confirmed was the peak in this year which seems epidemic. The radical decrease of cases in 2017 might show there were collective action of stakeholder on awareness creation, budget increment and implementation of malaria prevention and control strategies.

In the present study, Plasmodium species infections were higher in males than females. Since the study area is a rural town main agriculture is the main livelihood. Therefore, due to the fact that males are mainly engaged in agricultural activities and other large projects. They may spend the night outside with their properties and agricultural products. This can make them easily exposed to malaria vector.

Malaria cases were high in age group 15–45 years old and the distribution of mixed infection was almost similar in all age groups. This might be due to the fact that these age groups are active and productive forces they involved in agricultural and other activities which need to travel exposing places and spending the night while keeping their products. This was in agreement with reports from Kola Diba and Kombolcha [3, 13].

Among the factors that affect malaria transmission, seasonal variation has direct role. In the present study, malaria cases were increased from September to December. This period is considered as the major malaria transmission period in Ethiopia after the heavy rain in July and August. Similarly, there was an increment of malaria cases from May to July.

## Conclusions

Malaria is still among the major public health problems in the country. *Plasmodium falciparum* is the dominant species in the study area followed by *P. vivax*. Enhancing malaria detection and speciation skill of laboratory personnel and scaling up other malaria control and prevention activities are very crucial to significantly reduce the burden of malaria in the study area.
